# Detection of Bone Defects Using CBCT Exam in an Italian Population

**DOI:** 10.1155/2017/7523848

**Published:** 2017-10-17

**Authors:** Gianluca Gambarini, Gabriele Miccoli, Gianfranco Gaimari, Deborah Pompei, Andrea Pilloni, Lucila Piasecki, Dina Al-Sudani, Dario Di Nardo, Luca Testarelli

**Affiliations:** ^1^Department of Oral and Maxillofacial Sciences, Sapienza University of Rome, Rome, Italy; ^2^Department of Periodontics and Endodontics, University at Buffalo, Buffalo, NY, USA; ^3^Department of Restorative Dental Sciences, College of Dentistry, King Saud University, Riyadh, Saudi Arabia

## Abstract

**Background:**

The aim of this study was to evaluate the* in vivo* incidence and the location of fenestrations in a young Italian population by using CBCT.

**Materials and Methods:**

Fifty patients who had previously performed CBCT for planning third molar extraction or orthodontic therapy were selected for the study. No previous dental treatment had been performed on these patients. Overall, 1,395 teeth were evaluated. Root fenestrations were identified according to the definition of Davies and the American Association of Endodontists. Data was collected and statistically analyzed.

**Results:**

Fenestrations were observed in 159 teeth out of 1,395 (11% of teeth). In the lower jaw, we found 68 fenestrations (5%) and 91 in the maxilla (6,5%). Incisors were the teeth with the highest incidence of fenestrations.

**Conclusion:**

The relative common finding (11%) of fenestration supports the need for CBCT exams before any surgical/implant treatment to avoid complications related to the initial presence of fenestrations. CBCT was found to be an effective and convenient tool for diagnosing fenestration.

## 1. Background

Bone defects like dehiscence and fenestrations are common findings in natural dentition, being more frequent on the facial bone than on the lingual bone, and in the anterior teeth [1]. Fenestrations are isolated areas in which roots are denuded of bone and root surfaces are covered only by periosteum and overlying gingiva, but marginal bone is intact. Therefore, the marginal bone in fenestrations is intact [2].

Clinical diagnosis of fenestration is a challenge. Information derived from probing the gingival tissues in association with traditional radiographic diagnostic imaging provides guidelines for assessing the alveolar bone height and checking for the presence of bone defects, but they can very rarely detect fenestrations [3].

Moreover, fenestrations can also occur as an iatrogenic error in implant dentistry. In such cases, a fenestration is defined as a “vestibular or linguopalatal defect” or as an expression of a bone thickness deficiency that creates partial exposure of an implant that is completely surrounded by bone. This means that when buccal fenestrations occur, the implant partially protrudes through an opening in the intact bone plate, mostly on the buccal side. There fenestrations that occur in implant dentistry are divided into two cases [1]. A Class 1 fenestration is a minor penetration of the implant through the intact bone plate. A Class 2 fenestration is the formation of a convexity enclosing a “significant portion of the implant exposed.” The distinction between these two classes of fenestrations is important because they call for different repair measures.

Diagnosis of such fenestrations can be accomplished at two stages: (a) as early as implant placement during the surgery or (b) delayed during recall appointments of the implant patient. Since fenestrations can be a result of implant placement but can be also naturally present in the dentition; it is important to determine why and when the fenestration occurred and to differentiate between a natural finding and an iatrogenic error. A limited number of researches were conducted specifically on fenestrations, and most of them were conducted on skulls, with no relation to previous dental treatment, including extractions, periodontal surgery, and orthodontic therapy [3, 4]. Therefore, the aim of the present study was to evaluate the incidence and the location of fenestrations by using a CBCT exam in a young population, who had no previous dental treatment performed.

## 2. Materials and Methods

CBCT examinations were selected from Italian (Caucasian) patients between 18 and 30 years old, who had already performed the CBCT exams for third molar extraction or orthodontic purposes but had no dental treatment previously done. Criteria of inclusion were age lower than 30 years old, teeth present in both dental arches, without any previous orthodontic, restorative, surgical (extraction), or prosthodontic treatment.

Fifty CBCT exams were examined, for a total of 1,395 teeth (five agenesia were recorded). Images were obtained by using a CBCT exam (iCAT, Imaging Sciences International, Hatfield, PA, USA), with a single 360-degree rotation and 0,3 mm voxel dimension (exposition of 5,0 mA, 120 kV, 9,6 s for time exposition, and 0,3 of axial section). The sections used were 1 mm (FOV, 4 × 4 × 6 cm) and 1,28 mm thickness. This passes through the center of the root perpendicular to the alveolar crest. The long axis of the root has dictated the vertical orientation of the section. The measurements were performed with the maximum possible zoom.

**Figure 1:**
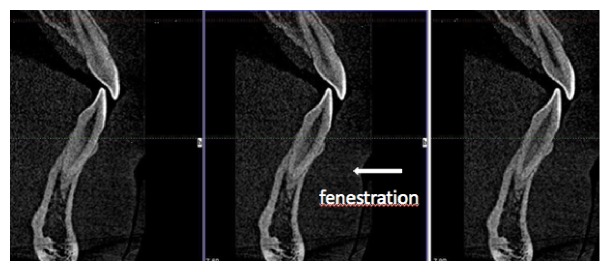
Cone-beam computed tomography (CBCT) images showing fenestrations in the area of the incisors.

Root fenestration (RF) was identified according to the definition of Davies et al. [5] and the American Association of Endodontists [6] as a tooth root protruding from a window-like opening or a defect in the alveolar bone without involvement of the alveolar margin. Three points should be emphasized in this definition. (1) A window-like opening or defect of the alveolar bone means that both the cortical bone and the cancellous bone are penetrated simultaneously, and the root either is in direct contact with the overlying mucosa or exposed to the oral environment. (2) The exposed root protrudes beyond the bone. (3) The exclusion of the alveolar margin emphasized to differentiate fenestration from dehiscence, which is the crest of buccal and/or lingual bone that lies at least 4 mm apical to the crest of the interproximal bone [5, 7, 8].  Figure 1 shows an example.

Data was analyzed using a Pearson chi-square test, performed using the Statistical Package for the Social Sciences (version 18.0; SPSS, Inc., Chicago, IL). The age, gender, number of fenestrations, and their location were displayed by frequency and percentage. The relations between the groups were analyzed by using the Pearson chi-square test. The level of significance was 5% (*P* < 0.05) and data was presented with 95% confidence intervals where applicable. All assessment was done by a double examiner to eliminate the interexaminer errors. All data regarding patient identification was kept confidential.

## 3. Results

The average age of patients was 24.5 years old, with 24 males (48%) and 26 females (52%).

In 588 teeth (42%), dehiscence, fenestrations, or both bone defects, were found. Fenestrations were present in 159 teeth out of 1,395, corresponding to the 11%.

**Figure 2:**
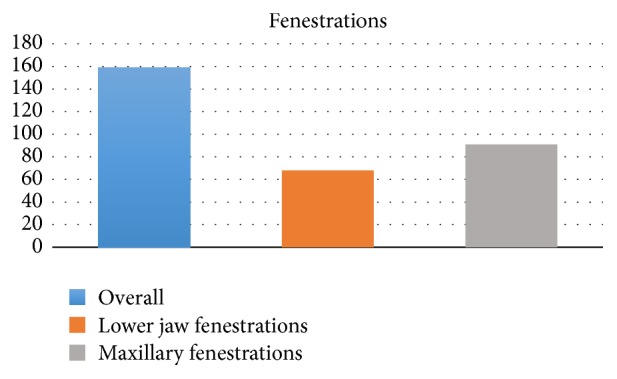
Incidence of fenestrations in maxilla and in mandible.

A significant difference was found (*P* = 0.0311) between the lower jaw presenting 68 fenestrations (5%) and the maxilla with 91 fenestrations (6,5%), as shown in Figure 2.

**Figure 3:**
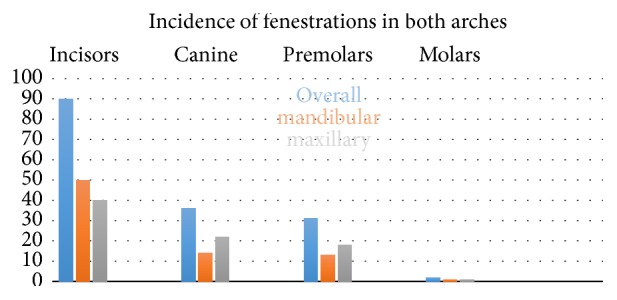
Incidence of fenestrations by tooth type.

Incisors were teeth with the highest incidence of fenestrations: 90 fenestrations (56%) were found in incisors, 50 in the maxilla (31%) and 40 in the lower jaw (25%); 36 cases (22%) were found in canines, 22 in the maxilla (13%) and 14 for the lower jaw (0.8%). Thirty-one (19%) fenestrations were found on premolars, 18 in the maxilla (11%) and 13 on the lower jaw (0.8%). The smallest incidence was found on molars in only 2 cases, 1 in the maxilla (0.1%) and 1 in the lower jaw (0,06%). Significant differences were found amongst these groups, with the exception of the canines compared to premolars (*P* = 0.068), as shown in Figure 3.

A significant difference was found (*P* = 0.0001) between 157 (99%) fenestrations observed in the labial-buccal side, compared with the two observed in the plagal-lingual side (1%). Multiple defects were significantly (*P* = 0,119) found more often (9.6%), when compared to patients showing only one defect (4%).

## 4. Discussion

Results of the present study showed that 11% of teeth have fenestrations as a natural finding, without any relation to previous dental treatments. This data confirms some, but not all, results from previous studies which had been performed on skulls, and consequently not in a young and healthy population. An in vivo study was performed to achieve more precise and detailed information. In cadavers skulls, due to differences in the composition of teeth and alveolar bone, the extent of degradation and damage differs between these two hard tissues. The alveolar plates in dried skulls, especially on the labial or buccal side, may be physically damaged more easily after exposure to air and soil, which may explain the higher prevalence of fenestration in skulls.

Fenestrations were present in 9.32% of teeth in skulls in the study conducted by Jorgic-Srdjak et al. in 1998 [9]; on the contrary, in the Nimigean et al. study [7], fenestrations were found in 69,56% of skulls. The latter results could have been influenced by surgical extractions, periodontal or other diseases [9–11].

In the present study, fenestrations were more common in the maxilla than in the mandible, which is consistent with previous reports [5, 7, 9–14]. However, differences can be found in the prevalence in some teeth. Previous studies reported the relative frequency of tooth type with fenestrations as follows: maxillary first molar, mandibular first molar, maxillary and mandibular canines, and mandibular lateral incisors [5, 7, 9, 11]. On the contrary, in the present study, fenestrations were found more common in incisors and rare in molars, probably due to the lack of any dental treatment, including extractions, in the molar area.

The present study confirmed that fenestrations were more frequent on the labial-buccal side than on the palatal-lingual. Elliot and Bower [15] reported only one unspecified lingual fenestration in a mandibular third molar. Edel [13] reported two lingual fenestrations in mandibular incisors because of inclined roots. Nimigean et al. [7] examined 3,646 teeth but reported no palatal or lingual fenestrations. A clear explanation of this phenomenon has not been reported, but it can be hypothesized that since most fenestrations occur in maxillary teeth, this might be a potential contributing factor, as many teeth in this arch have root tips inclined to the labial-buccal [7].

The traditional method of investigating the prevalence and morphology of fenestrations has been on dry human skulls. Visual examination and direct measurement make this method highly accurate and reliable; however, disadvantages are that studies of dried skulls offer no clinical information and no dental history, and the method can never be applied to clinical surgical diagnosis [16]. On the contrary, both in vivo and ex vivo studies have indicated that CBCT may be a useful and more practical clinical tool to detect these defects. The low dose of radiation and excellent image quality of CBCT compared with conventional CT makes CBCT the ideal means for the diagnosis of fenestration defects [17]. The use of CBCT allows clinicians to examine the form and the size of the alveolar bone without the disadvantages of common radiography. These images are not subject to distortions or overlaps. In the present study, CBCT scans were used to evaluate alveolar bone defects with axial and transverse sections. Fenestration was easily detected, confirming the conclusions of the Bayat et al. study [18]. CBCT currently represents the gold standard to evaluate fenestrations and dehiscences furcation lesions, instead of the common radiology.

To date, only few investigations have taken advantage of CBCT to study fenestration. The studies of Leung et al. [19], Ising et al. [20], and Patcas et al. [2] indicated that CBCT is a reliable, accurate, and noninvasive method for diagnosing fenestrations in the clinical setting and for investigating its prevalence. Visualizing dehiscences and fenestrations is not possible with traditional two-dimensional (2D) radiographs because of superimposition. CBCT allows the visualization of these defects with more accurate three-dimensional (3D) images [21–24].

Of course, unpredictable occurrences, such as iatrogenic errors during implant placement, cannot be eliminated by any precautionary measures, but a preoperative CBCT can help to ensure proper placement in implant angulation and to ensure a proper distance from the adjacent tooth and the external bone surface.

Other studies [25] used CBCT to compare the correlations between the presence of fenestrations and malocclusions. Significant differences in the presence of fenestration were found among subjects with skeletal Class I, Class II, and Class III malocclusions. Fenestrations had greater prevalence in the maxilla and were more common in Class II. A study on Chinese population [26] found a higher presence of fenestrations (31,93% of teeth) in Class III patients. The tooth site which was most commonly affected was lower canine, while the least was upper central incisor. All these studies indicated that malocclusions are potential factors for bone defects and fenestrations. In the present study, it was not possible to check this correlation in an Italian population because half of the patients had been previously scanned for surgical purposes and no orthodontic parameters had been registered.

## 5. Conclusion

Fenestrations of the buccal bone are unpredictable anatomical findings, difficult to diagnose by traditional clinical and radiographic technique. It is important to diagnose these defects before any surgical, implant, or orthodontic therapy, since undetected fenestrations may adversely affect the clinical outcome of these treatments. The relative common finding of fenestrations in an Italian population supports the need of a preoperative CBCT exam to accurately diagnose the initial presence of fenestrations, for a more precise and reliable surgical approach and implant placement.
